# Post-stroke low-frequency whole-body vibration improves cognition in middle-aged rats of both sexes

**DOI:** 10.3389/fnagi.2022.942717

**Published:** 2022-08-17

**Authors:** Nadine Kerr, Juliana Sanchez, William Javier Moreno, Ofelia E. Furones-Alonso, W. Dalton Dietrich, Helen M. Bramlett, Ami P. Raval

**Affiliations:** ^1^Department of Neurological Surgery, Leonard M. Miller School of Medicine, University of Miami, Miami, FL, United States; ^2^Bruce W. Carter Department of Veterans Affairs Medical Center, Miami, FL, United States; ^3^Peritz Scheinberg Cerebral Vascular Disease Research Laboratory, Department of Neurology, Leonard M. Miller School of Medicine, University of Miami, Miami, FL, United States

**Keywords:** sex differences, low-frequency vibration, inflammation, vascular endothelial growth factor, irisin, fibronectin type III domain 5, stroke, rehabilitation

## Abstract

Low-frequency whole-body vibration (WBV; 40 Hz), a low impact form of exercise, intervention for a month following moderate transient middle-cerebral artery occlusion (tMCAO) reduces infarct volume and improves motor function in reproductively senescent, middle-aged female rats. Since post-stroke cognitive decline remains a significant problem, the current study aims to investigate the efficacy of WBV in ameliorating post-tMCAO cognitive deficits and to determine the underlying putative mechanism(s) conferring benefits of WBV in middle-aged rats. Middle-aged rats of both sexes were randomly assigned to tMCAO (90 min) or sham surgery followed by exposure to either WBV (twice a day for 15 min each for 5 days a week over a month) or no WBV treatment groups. Following the last WBV treatment, rats were tested for hippocampus-dependent learning and memory using a water maze followed by harvesting brain and blood samples for histopathological and inflammatory marker analyses, respectively. Results show that post-tMCAO WBV significantly lessens cognitive deficits in rats of both sexes. Post-tMCAO WBV significantly decreased circulating pro-inflammatory cytokines and increased serum levels of irisin, a muscle-derived hormone that may play a role in brain metabolism and inflammation regulation, which suggests putative beneficial mechanisms of WBV.

## Introduction

Stroke is the leading cause of disability worldwide, and most stroke survivors suffer functional and cognitive deficits (Barba et al., 2000; Stephens et al., 2004; Levine et al., 2015). Decline and impairment of cognition also predict the onset of physical frailty, and vice versa (Gross et al., 2016). Effective rehabilitation interventions initiated early after stroke can enhance the recovery process and minimize functional disability. Early rehabilitation may also mitigate the risk of a subsequent stroke, reduce cognitive decline, and improve the overall quality of life after stroke. The duration of the stroke rehabilitation depends on stroke severity. Most stroke survivors require some form of long-term stroke rehabilitation, but clinical studies report that compliance with such long-term stroke rehabilitation is very poor (Morris and Williams, 2009; Cohan et al., 2019; Levy et al., 2019). A wide range of individual factors may affect stroke patient participation in physical therapy, including stroke severity, preexisting and comorbid conditions, motivation, fatigue, and depression. Additionally, long-term stroke rehabilitation may not be covered by insurance. Therefore, identifying novel, low-cost effective rehabilitation intervention(s) to mitigate post-stroke cognition remains a high priority in the field and is the main goal of the current study.

In recent years, whole body vibration (WBV) emerged as a cognitive-enhancing therapy, especially in persons unable to perform active/aerobic forms of exercise (Regterschot et al., 2014). The aerobic exercise improves cardiovascular adaptations and increases peak oxygen consumption without significantly changing strength (Lambert and Evans, 2005; Villareal et al., 2017; Pahl et al., 2018). On the contrary, WBV emerged as an alternative exercise modality for strength training. The WBV-induced strength training improves neuromuscular adaptations and thus increases strength without significantly changing peak oxygen consumption (Lambert and Evans, 2005; Villareal et al., 2017; Pahl et al., 2018). Studies provide evidence that WBV improves body composition and muscle strength (Delecluse et al., 2003; Roelants et al., 2004; Prisby et al., 2008; Fontana et al., 2022). Furthermore, WBV enhances force and power in skeletal muscle, and at the molecular level, it leads to the formation of reactive oxygen species generation similar to that following treadmill exercise (Lopez-Cervantes et al., 2022). The moderate level of reactive oxygen species generation confers muscular adaptation via mitochondrial biogenesis. Therefore, WBV therapy has the potential to induce both mechanical and metabolic adaptive responses.

In several recent studies, physical activity of skeletal muscle resulted in the release of extracellular vesicles (EVs) into circulation (Fruhbeis et al., 2015). These extracellular vesicles are involved in cell-to-cell communication and may bring myokines and cytokines (peptides that regulate metabolism) to distal organs, such as the heart and brain (Molteni et al., 2002; Bei et al., 2017). One myokine, irisin, is secreted from muscles in response to exercise, and WBV also increases irisin levels in the human body following exercise (Huh et al., 2014). In a clinical study, a group of healthy, untrained females endured bouts of acute WBV exercise for a period of 6 weeks. At the end of the 6-week exercise period, the levels of irisin increased significantly (twofold) compared to irisin levels in females who did not participate in WBV exercises (Huh et al., 2014).

Whole body vibration is a method that amplifies baseline irisin levels in humans (Rees et al., 2009; Wrann, 2015). Irisin is a cleavage protein of fibronectin type III domain 5 (FNDC5) and irisin increases following exercise (Wrann, 2015; Leal et al., 2018). In mice, exercise enhances FNDC5 expression and likely irisin levels in the hippocampus of mice (Wrann, 2015). Irisin treatment reduces brain infarct volume, neurological deficit scores, and neuronal injuries in mice subjected to MCAO (Asadi et al., 2018). Post-stroke high-intensity interval training reduced ischemic brain damage and upregulated pTrkB (a major cascade of BDNF actions) and FNDC5 expression in the cortex of rats (Pin-Barre et al., 2021), which underscores its role in neuronal survival, hippocampal neurogenesis, synaptic plasticity, and functional recovery (Liu and Nusslock, 2018; Cohan et al., 2019; Luo et al., 2019). Irisin treatments have also demonstrated improved memory and synaptic plasticity in mouse models of Alzheimer’s disease (Lourenco et al., 2019). Important to this study, in a tMCAO rodent stroke model, irisin levels and skeletal muscle expression of FNDC5 were decreased in rats (Li et al., 2017).

Our published studies demonstrated that (1) low-frequency whole-body vibration (WBV) therapy after spinal cord injury improved selected biomarkers of bone turnover and gene expression and reduced osteoclastogenesis (Bramlett et al., 2014), and (2) 1 month-long WBV therapy after moderate transient middle cerebral artery occlusion (tMCAO) significantly reduced infarct volume and improved motor function after tMCAO in middle-aged female rats (Raval et al., 2018). Furthermore, post-tMCAO WBV intervention greatly reduced inflammation and significantly increased brain-derived neurotrophic factor and improved functional activity in middle-aged female rats (Raval et al., 2018). Therefore, the current study aims to test the efficacy of post-tMCAO WBV therapy in ameliorating cognitive deficits in reproductively senescent middle-aged female and age-matched middle-aged male rats. We hypothesize that WBV therapy improves post-stroke outcomes via increasing circulating irisin.

## Materials and methods

All animal procedures were carried out in accordance with the Guide for the Care and Use of Laboratory Animals published by the U.S. National Institutes of Health and were approved by the Animal Care and Use Committee of the University of Miami. Results are reported according to the Animal Research: Reporting *in Vivo* Experiments guidelines to the best of our knowledge.

**FIGURE 1:**
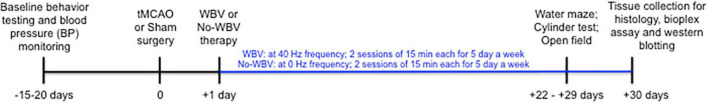
Experimental design. Middle-aged rats of both sexes were included in the study. The blood pressure and baseline behavior testing were performed on rats over the period of 2–3 weeks. Rats were then exposed to moderate tMCAO (90 min) or sham surgical procedure. A day after tMCAO, rats were randomly assigned to two groups. One group of rats were sacrificed for tissue collection and another group of rats were randomized for WBV or No-WBV therapy. During the period of 1-month survival, blood pressure of animals was monitored twice per week. Either on 22nd or 23rd day after induction of tMCAO, rats were tested for hippocampus-dependent learning and memory over the period of 7 days, followed by cylinder and open field tests. On day 30th, rats were randomized into two groups: one group of rats were sacrificed for blood and tissue collection for biochemical analysis, and brain tissue from the other group of rats was allocated for histopathological analysis.

Retired breeder female (10–13 months; 280–380 g; *n* = 60) or age-matched male (*n* = 52) Sprague–Dawley rats were used to perform the experiments (Figure 1) and details for rats used are provided in Supplementary Table 1. Estrous cycles of retired breeder rats were monitored by examining daily vaginal smears as described in our earlier publications (de Rivero Vaccari et al., 2015). Rats that persisted in any one estrous cycle stage for 7 days were considered acyclic. Reproductively senescent, middle-aged female animals were included in the study if they remained in constant diestrus (de Rivero Vaccari et al., 2015). We used middle-aged-matched male rats because, in contrast to females, reproductive aging in male rats resulting in decreased fertility occurs much later in lifespan. Rats of both sexes were handled for 2–3 weeks prior to induction of tMCAO for baseline blood pressure monitoring and behavioral testing followed by random assignment to tMCAO or sham surgical procedures.

Rats of both sexes were exposed to tMCAO for 90 min or sham surgery as previously described (Raval et al., 2018). Temperature probes were inserted into the rectum and the left temporalis muscle, and separate heating lamps were used to maintain rectal and cranial temperatures at 37°C to 37.5°C. The right common carotid artery (CCA) was exposed through a midline neck incision and was freed from surrounding nerves and fascia and carefully dissected from its bifurcation to the base of the skull. The occipital artery branches of the external carotid artery (ECA) were then isolated, and these branches were dissected and ligated. The ECA was dissected further distally and ligated along with the terminal lingual and maxillary artery branches, which were then divided. The internal carotid artery (ICA) was isolated and carefully separated from the adjacent vagus nerve and the pterygopalatine artery. The middle cerebral artery (MCA) was then occluded by suture insertion. Once the animals awakened from anesthesia, rats were returned to their cages. The suture was gently withdrawn after the 90-min occlusion period. In parallel, we also performed sham surgery where rats were exposed to similar surgical procedures including exposure to anesthesia for the same duration, right carotid artery, ECA, and ICA dissections except that the MCA was not occluded by suture. Following tMCAO, rats were randomly assigned to WBV or No-WBV treatments. The overall mortality was 10% (12/112) within 24 h of surgical intervention. The mortality did not significantly differ among the male and female rats.

### Low-frequency whole-body vibration therapy

One day after the tMCAO, animals were randomly assigned to (1) a WBV intervention group or to (2) a No-WBV control group. Animals randomized to the WBV group underwent 30 days of treatment performed twice daily for 15 min (once in the morning between 8:00 and 10:00 a.m. and once in the afternoon between 2:00 and 4:00 p.m.) each session for 5 days each week. The vibration device (Soloflex, Hillsboro, OR) was programmed in order to achieve a frequency of vibration within a range of about 40 Hz similar to that used in clinical studies (Xie et al., 2006; Herrero et al., 2011; Wysocki et al., 2011). The duration and frequency of sessions were selected based on our publication (Bramlett et al., 2014), where we demonstrated the ability of WBV to improve selected biomarkers of bone turnover and gene expression and to reduce osteoclastogenesis after spinal cord injury. To avoid any confounding associated with handling or exposure to vibration platform, rats allocated to both No-WBV and WBV groups were handled and placed on the vibration table in a similar fashion. Animals randomized to the No-WBV group were placed on the vibration platform for 15 min (duration of WBV treatment) without activation of the platform. Importantly, the duration spent in the chamber or with vibration and handling was the same between both control (No-WBV) and WBV groups. To provide WBV intervention or No-WBV, rats were placed in a plexiglass box that contained four chambers. Each rat was placed into a chamber in random order from one session to the next to avoid any bias due to chamber placement. The vibration parameters were measured in each chamber and differences in these parameters between the chambers were negligible. No animals were excluded for their inability to tolerate WBV.

### Blood pressure monitoring in middle-aged female rats

Hemodynamic measures were evaluated using the tail cuff method (CODA 2 system; Kent Scientific). For 5 consecutive weeks, 10 measurements were taken for each animal and the mean value was reported in Supplementary Figure 1. These parameters include measurements for systolic pressure, diastolic pressure, and average blood pressure.

### Monitoring of activity levels in middle-aged female rats

Rat activity levels were tested in an open field (43.5 × 21.5 cm) with four white arenas using a video tracking system (EthoVision). Rats were placed in each arena, and 5-min recordings were taken prior to and after completion of WBV/No-WBV treatment. The total duration spent in the center of the open field was measured.

### Cylinder test in middle-aged female rats

Animals were evaluated for spontaneous forelimb placement in a transparent Plexiglas cylinder (20 cm diameter × 30 cm height) for 5 min. The cylinder geometry encouraged vertical exploration (Blaya et al., 2014). Animals were first evaluated for baseline behavior in the cylinder test at 1–3 days prior to tMCAO, then were re-evaluated at 1-month post-tMCAO. The number of times the right or left forelimb made contact with the wall while the animal was rearing was counted. Asymmetry index was calculated by dividing the number of contralateral (left) forelimb touches by total forelimb touches.

### Hippocampal-dependent learning and memory in middle-aged rats of both sexes

Rats exposed to tMCAO and WBV treatment were monitored for memory and spatial learning using the Morris Water Maze (Bramlett et al., 1999). In this behavioral procedure, the rats were placed inside a small circular pool (122 cm diameter; 60 cm deep; filled with opaque water at 21°C) with a platform located in the north-east corner. Animals were tested with a spatial reference memory task using a water maze (4 trials/day, 4 min inter-trial interval, 60 s trial duration). At 24 h after the final testing day, animals were subjected to a probe trial (30 s trial duration) with the platform removed to assess retention and evaluate the search strategy. Next, to assess spatial working memory, the animals were tested for 2 days in the water maze with a hidden platform that remained invariant only during each pair of trials. The ability to find the platform on the second trial was compared to the first trial performance with an inter-trial interval of 5 s. The animal’s movement was videotaped with a CCD camera and analyzed with the Ethovision software program as described previously (Raval et al., 2013). The memory and learning capabilities of rats were tested at the end of the WBV/control exposure. At the end of behavioral testing (7-day paradigm), rats were euthanized, and tissues were collected for histopathology assessments. Infarct volumes are presented in Supplementary Figure 2.

### Bio-Plex assay in serum of middle-aged rats of both sexes

Separate cohorts of rats of both sexes exposed to tMCAO and tMCAO + WBV/No-WBV were sacrificed at 24 h and 1 month after tMCAO respectively, for brain tissue and blood/serum collection (details of euthanasia methods are provided in the Supplementary Data). Serum samples were used for the Bio-Plex assay. The Bio-Plex immunoassays are volume-sparing, requiring a total of only 50 μl of serum to run each sample in duplicate. Serial dilutions of standards representing each analyte and an in-house control plasma sample were run in duplicate on each 96-well assay plate. Fluorescence intensities of each analyte-specific immunoassay bead were analyzed on the Bio-plex 200 System with HTF (Bio-Rad). Raw data were captured using Bio-Plex Manager Software 6.1. A concentration of individual immune factors in each sample was interpolated from standard curves using a five-parameter, weighted logistic regression curve equation in Bioplex Manager Software 6.1. We used the Bio-Plex Pro™ Rat Cytokine 24-plex Assay (cat #171K1001M from Bio-Rad) and additional data are presented in Supplementary Tables 2–5. The 24 analytes chosen for Bio-Plex assay are based on functional immune markers categories, which are associated with inflammatory and immune-mediated injury in cerebrovascular and neuropsychiatric disorders, including dementia.

### Measurement of irisin levels in the serum of middle-aged female

The serum derived from middle-aged female that underwent tMCAO followed by WBV or No-WBV was used to measure irisin levels using an ELISA Kit (Novus Biologicals, Catalog # NBP2-67959).

### Isolation of extracellular vesicles from serum of middle-aged female

The sera derived from middle-aged females that underwent tMCAO followed by WBV or No-WBV were used to isolate EV. Serum EV were isolated using the Total exosome isolation from serum kit (Invitrogen) as described in a previous publication (Kerr et al., 2018). Briefly, 100 μl of each sample was centrifuged at 2,000 × g for 30 min and the supernatant was then incubated with 20 μl of Total exosome isolation reagent for 30 min at 4°C followed by centrifugation at 10,000 × g for 10 min at room temperature. Supernatants were then discarded, and the pellet was re-suspended in 50 μl of PBS and 100 μl of lysis buffer. The concentration and size distribution of the isolated exosomes were analyzed by a NanoSight NS300 system (Malvern Instruments Company, Nanosight, and Malvern, United Kingdom).

### Western blotting

The protein content in homogenized cortical tissue and serum-derived extracellular vesicles (EV) was assessed and proteins were separated by 12% stain-free SDS-PAGE as described in previous publications (Raval et al., 2009). Proteins were subsequently transferred to a Polyvinylidene difluoride (PVDF) membrane and incubated with primary antibodies against anti-FDNC5 (1:1,000; Rabbit anti-FDNC5, Catalog # ab174833, abcam) and total protein is presented as loading control (Raval et al., 2009).

### Statistical analysis

The data are shown as mean ± SD. All the data were analyzed using a one-way ANOVA followed by Tukey’s multiple comparison tests for two or more groups. Data were normally distributed, using a D’Agostino–Pearson test for normality. A *p* < 0.05 was considered statistically significant.

## Results

### Post-transient middle-cerebral artery occlusion whole body vibration therapy improves sensory motor function in middle-aged female rats

**FIGURE 2:**
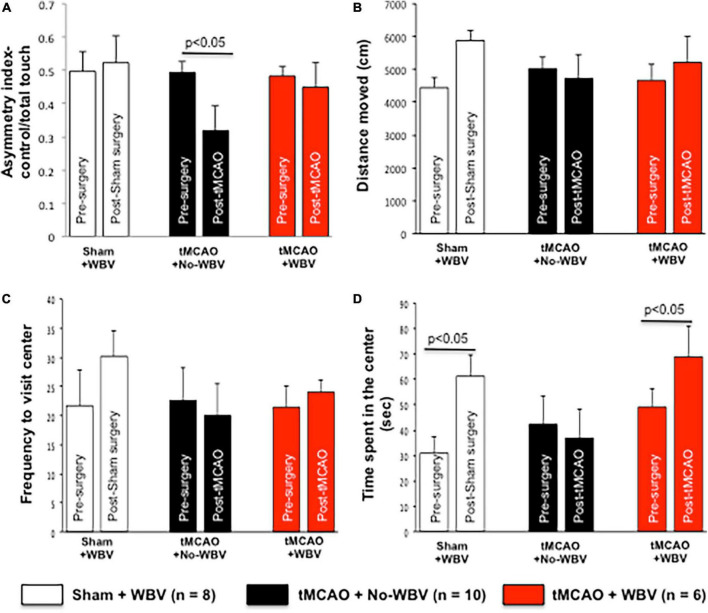
**(A–D)** Post-tMCAO WBV therapy improves sensory motor function and increases open field activity in middle-aged female rats. Rats were evaluated for spontaneous forelimb placement and the number of times the right or left forelimb made contact with the wall while the animal was rearing was counted. Asymmetry index was calculated by dividing the number of contralateral (left) forelimb touches by total forelimb touches. Data presented in panels **(B–D)**, shows open field activity test data of middle-aged female rats. Data presented shows total distanced moved, frequency to visit center and time spent in the center. Activity was monitored 1–3 days prior to induction of tMCAO and 1 month after WBV/No-WBV exposure. Data presented in Panel **(D)** demonstrate that post-tMCAO No-WBV group spent significantly less time in the center of the open field, while post-tMCAO WBV treated rats spent significantly more time in the center as compared to the No-WBV group.

We hypothesized that post-tMCAO improves motor deficits, and in order to evaluate sensorimotor deficits, we utilized the cylinder test. This task quantifies spontaneous asymmetrical forelimb use and reliably detects deficits in models that produce substantial unilateral damage, which occurs after focal cerebral ischemia (Hatinen et al., 2008; Blaya et al., 2014). For each rat, the asymmetry index at 1-month post-tMCAO was normalized to the baseline asymmetry index to account for any pre-operative bias (Schaar et al., 2010). At 1-month post-tMCAO, WBV treated animals had a significant increase in asymmetry index, indicating improvement that was absent in No-WBV-treated rats (Figure 2A).

We conducted open field activity tests to monitor alterations in animal activity following tMCAO and to study the impact of WBV in female rats. The results demonstrate that prior to tMCAO-induction, the time spent in the center of the open field is different amongst groups; however, the observed differences are not significant. Furthermore, the results demonstrated no significant difference between sham and post-tMCAO plus No-WBV activity measurements of distance traveled or frequency to visit the center of the field (Figures 2B,C). Post-tMCAO, the No-WBV group spent significantly less time in the center of the open field. In contrast, post-tMCAO WBV treated rats spent significantly more time in the center as compared to the No-WBV group (Figure 2D).

### Post-transient middle-cerebral artery occlusion whole body vibration therapy improves spatial learning and memory in middle-aged rats of both sexes

**FIGURE 3:**
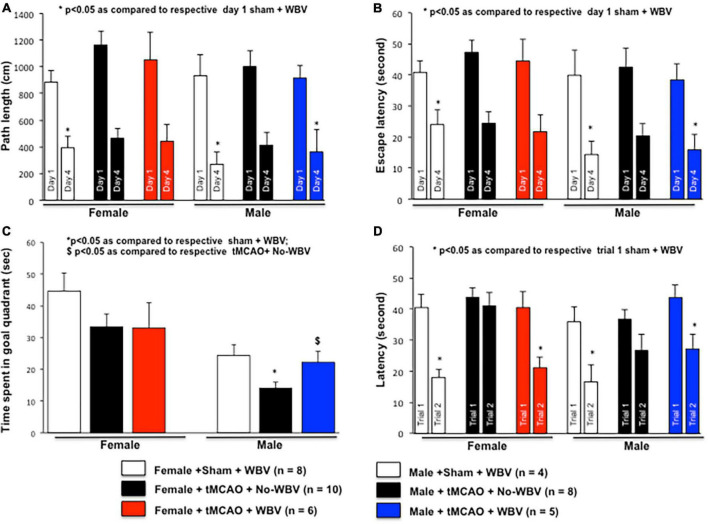
Effect of post-tMCAO WBV therapy on spatial learning and memory in middle-aged rats of both sexes. The hippocampus-dependent learning and memory of rats of both sexes were evaluated using the water maze test starting the 22nd or 23rd day post-tMCAO. The simple placement task was a 4-day test in which the hidden platform was placed in the north-east quadrant of the pool. Animals received four 60 s acquisition trials per day for 4 days with an intertrial interval of 4–6 min. The dependent measures for this test are the path length and latency required to find the platform. Panels **(A)** and **(B)** depicts normalized path length and latencies to day 1 of sham treated with WBV. The results show a significant decrease in path length and latency in finding the hidden platform on the 4th day in sham +WBV, No-WBV, and WBV treated rats of both sexes. Next, a probe trial was conducted with the platform removed and the time spent in the goal quadrant, where the platform used to be, was measured. The data presented in panel **(C)** demonstrated that the animals spent significantly less time in the goal quadrant after tMCAO as compared to the sham group. We observed a significant difference in percent time spent in the goal quadrant between WBV or No-WBV groups in male but not in female rats. Lastly, animals were tested for their ability to find a new platform placed in a different location of the pool per paired trials as an indicator of working memory. Panel **(D)** depicts normalized latencies to trial 1 of sham treated with WBV. The data demonstrated that rats of both sexes treated with WBV took significantly less time to locate a new platform as compared to No-WBV.

Cognitive impairments following stroke are recapitulated in animal models, and emerging studies demonstrate that neurodegeneration occurs in the rat hippocampus and striatum after MCAO (Butler et al., 2002; Di Filippo et al., 2008; Wattanathorn et al., 2011). Since, we observed functional improvements following WBV in tMCAO-exposed female rats both in a previously published study (Raval et al., 2018) and in the results, we further decided to investigate hippocampal neuronal survival using histology at 1 month after tMCAO. We tested the hypothesis that post-stroke WBV improves cognition in female and male rats. Rats exposed to tMCAO/sham followed by WBV/No-WBV treatment (Figure 1), were evaluated using the water maze test starting the 22nd or 23rd day post-tMCAO. Figures 3A,B depict absolute distance and latency. The results show a significant decrease in both distance traveled and latency in finding the hidden platform on the fourth day in sham, No-WBV, and WBV-treated female and male rats and suggest no difference in learning to find the platform using spatial clues among the different groups.

After a learning period of 4 days, a probe trial (30 s duration) was given with the platform removed and the time spent in the goal quadrant, where the platform used to be, is measured. The data presented in Figure 3C demonstrates that the animals spent significantly less time in the goal quadrant after tMCAO as compared to the sham group. We observed a significant difference in percent time spent in the goal quadrant between WBV or No-WBV groups in male but not in female rats, suggesting a possible sex difference in the effects of WBV. On day 7 of water maze paradigm, animals were tested for their ability to find a new platform placed in a different location of the pool per paired trials as an indicator of working memory. Figure 3D depicts normalized latencies to trial one of shams treated with WBV. The data demonstrate that rats of both sexes treated with WBV took significantly less time to locate a new platform as compared to No-WBV.

### Sex differences in post-transient middle-cerebral artery occlusion serum levels of interleukin 1β

**FIGURE 4:**
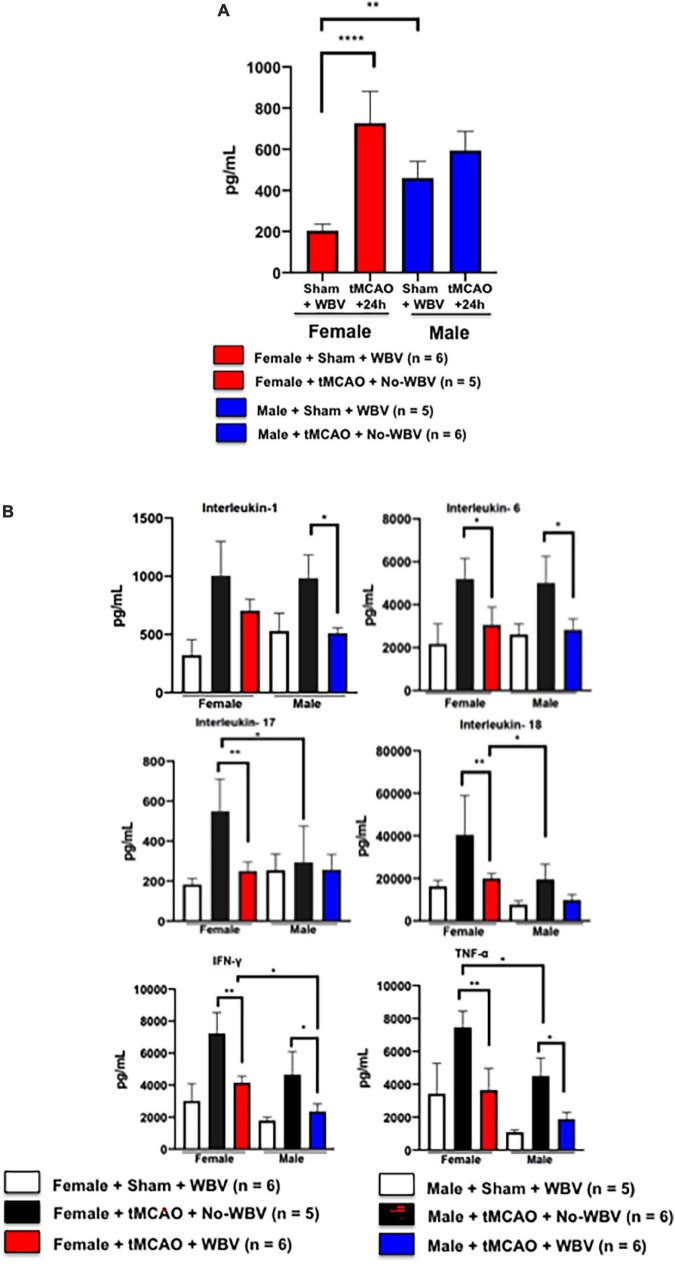
**(A)** Serum IL-1β increases in middle-aged female rats 24 h after tMCAO. Data presented in the figure shows a significant increase in serum IL-1β levels in middle-aged female but not in male rats. **(B)** Post-tMCAO WBV significantly decreases circulating pro-inflammatory cytokines in middle-aged female and male rats. Data presented in the figure shows that in animals of both sexes, post-stroke WBV significantly reduced circulating pro-inflammatory cytokines IL-6, IFN-gamma, and TNF alpha as compared to the respective No-WBV group. IL-1 remained high in females in contrast to males after WBV treatment. In males, IL-17 and IL-18 did not show changes similar to those in female rats either treated with No-WBV or WBV. *p < 0.05, **p < 0.01, ****p < 0.001.

Our published study demonstrated that inflammasome activation and the pro-inflammatory cytokine IL-1β were significantly higher in the brain and serum of reproductively senescent females as compared to their younger counterparts and male rats (Raval et al., 2019). Therefore, we investigated circulating IL-1β levels in middle-aged rats of both sexes 24 h after induction of tMCAO. Using the Bio-Plex assay, we observed a significant increase in serum IL-1β levels in female but not in male rats (Figure 4). These data confirm epidemiological and animal data from various laboratories that at the transition to peri-menopause or reproductive senescence respectively, females show increased systemic inflammation and suffer severe ischemic damage as compared to young female or male counterparts (Raval et al., 2019; McCarthy and Raval, 2020).

### Post-stroke whole body vibration reduces circulating pro-inflammatory cytokines in rats of both sexes

We have demonstrated that WBV treatment reduced the expression of IL-1β in the brains of female rats after stroke (Raval et al., 2018). Others have also demonstrated that WBV treatment has an effect on various systemic cytokines including tumor necrosis factor (TNFα), interleukins IL-10, IL-1β, IL-6, IL-17, IL-18, and vascular endothelial growth factor (VEGF) in humans (Simao et al., 2012; Lage et al., 2018). Using the Bio-Plex Pro™ Rat Cytokine 24-plex (cat #171K1001M; selected 24 analytes are functional immune markers) assay, we observed reduced circulating pro-inflammatory cytokines in the serum of post-tMCAO WBV-treated female and male rats (Figure 4B). In animals of both sexes, post-stroke WBV significantly reduced circulating pro-inflammatory cytokines IL-6, IFN-gamma, and TNF alpha as compared to the respective No-WBV group. IL-1 remained high in females in contrast to males after WBV treatment. In males, IL-17 and IL-18 did not show changes similar to those in female rats either treated with No-WBV or WBV. Unlike the aged-female group, IL-1α, a proinflammatory cytokine that has been implicated in neuroinflammatory progression post-stroke (Denes et al., 2011), was significantly decreased in male rats treated with WBV. Our data indicate that males and females respond to stroke therapies differently and that the immune response plays a role in this difference (Dotson and Offner, 2017).

### Post-stroke whole body vibration increases circulating vascular endothelial growth factor in animals of both sexes

**FIGURE 5:**
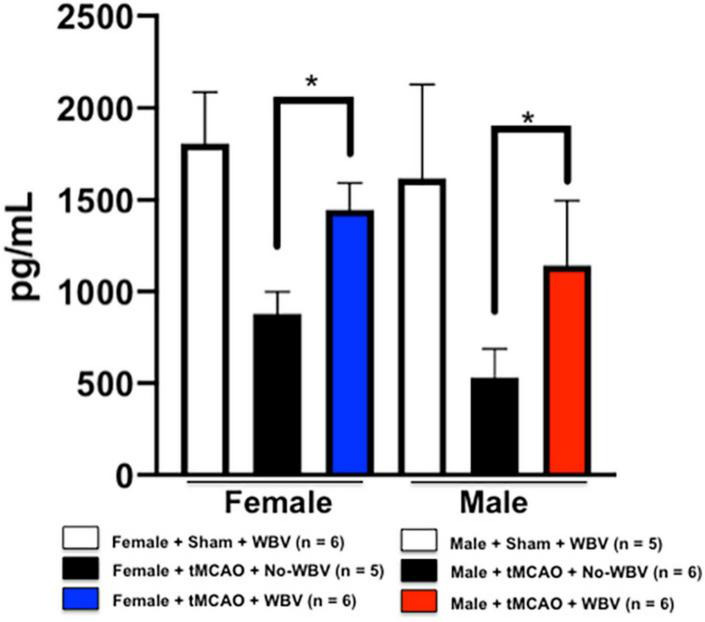
Post-stroke WBV increases serum VEGF levels in rats of both sexes. Data presented in the figure shows the significant increases in circulating VEGF levels in the WBV treated group compared to No-WBV treated middle-aged rats of both sexes. *p < 0.05.

Vascular endothelial growth factor is known to increase after stroke. It is known to promote neuroprotection, neurogenesis, angiogenesis, and brain vessel repair (Greenberg and Jin, 2013). We observed significant increases in circulating VEGF levels in the WBV-treated group compared to No-WBV treated rats of both sexes, suggesting its role in angiogenesis and systemic repair post-stroke (Figure 5).

### Post-transient middle-cerebral artery occlusion whole-body vibration increases irisin protein levels in the serum and cortical penumbra of middle-aged female rats

**FIGURE 6:**
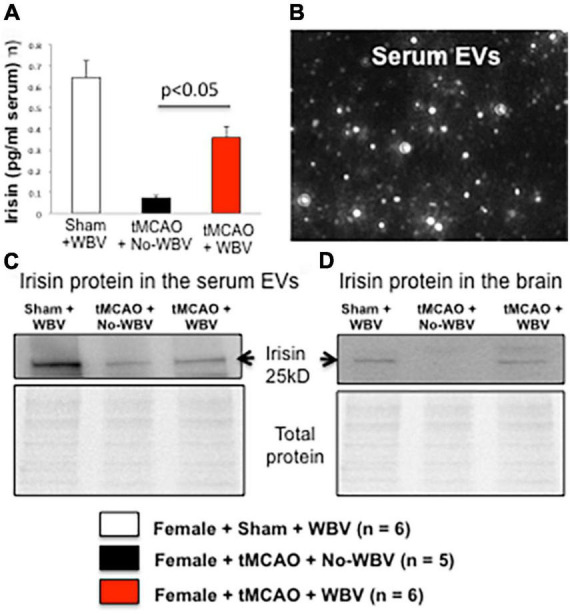
**(A)** Irisin levels in serum. **(B)** Image showing isolated exosomes using NanoSight NS300 system. **(C,D)** Post-stroke WBV increases irisin protein levels in serum-derived EVs and in cortex of sham + WBV and tMCAO + WBV groups but not in no-WBV group.

Several recent studies have shown that following physical activity, skeletal muscle releases extracellular vesicles (EVs) into the circulation (Fruhbeis et al., 2015). These extracellular vesicles are involved in cell-to-cell communication and may bring myokines and cytokines to distal organs, such as the heart and brain (Molteni et al., 2002; Bei et al., 2017). One myokine, irisin, is secreted from muscles in response to exercise, and WBV increases irisin levels in the human body following exercise (Huh et al., 2014). Therefore, we tested the hypothesis that post-stroke WBV increases circulating irisin and EVs containing irisin, and these EVs increase the availability of irisin protein levels in the brain and serum. Middle-aged female rats treated with WBV showed higher levels of serum irisin than the No-WBV group (Figure 6). Western blot analysis demonstrated that WBV treatment increases irisin protein levels compared to No-WBV group in serum-derived EVs and in cortices of female rats.

## Discussion

The most important outcome of the current study is that post-tMCAO low-frequency WBV therapy for a month significantly improves hippocampus-dependent cognition in middle-aged rats of both sexes. Using the spatial probe, the most sensitive test for post-stroke learning deficit (Lyden and Lonzo, 1994), we observed some sex differences. For example, we saw significant increases in percent time spent in the goal quadrant between WBV or No-WBV groups in male but not in female rats, suggesting a sex difference in the effects of WBV. We also observed a significant improvement in motor function and open field activity in female rats. One potential reason for the improved behavioral outcome is that WBV might decrease the infarct volume. Our data, however, showed a trend but no significant reduction in the infarct volume in middle-aged female rats. In contrast, in a previous published study, post-tMCAO WBV therapy reduced infarct volume in middle-aged female rats. The difference between our two studies is the duration of ischemia. In the previous study, rats were exposed to 60 min of mild ischemia. In contrast, the current study used 90 min of moderate tMCAO. These data suggest that post-tMCAO, WBV-induced reduction in the infarct volume depends on the severity/duration of ischemia and remains efficacious in improving motor and cognitive outcomes. The underlying mechanisms by which WBV improves motor and cognitive deficits after tMCAO remain a question, and a possible pathway could be via systemic effects similar to those noted following physical exercise.

Studies have demonstrated that physical exercise induces systemic inflammation, which improves cognition (Svensson et al., 2015; Norman et al., 2018). The cross talk between local and systemic inflammatory mediators released after an ischemic episode governs the overall ischemic outcome. In the current study, we observed that post-tMCAO WBV significantly reduced circulating pro-inflammatory cytokines IL-1β, IL-6, IL-18, IFN-γ, and TNF-α as compared to the respective No-WBV group. However, males and females respond differently to post-tMCAO WBV therapy and trigger different immune inflammatory factors. In general, systemic inflammation eventually subsides and injured tissue undergoes structural and functional reconstruction, which requires a variety of growth factors including VEGF (Ergul et al., 2012). Here we present data depicting significant increases in circulating VEGF levels in the rats of both sexes following WBV-treatment compared to No-WBV-treated group. Systemic VEGF levels increase post-tMCAO in rats, as well as angiogenesis and observed blood vessel repair which suggest CNS and systemic angiogenesis post-tMCAO in rats of both sexes (Greenberg and Jin, 2013). Future studies, however, will be necessary to confirm the effect of WBV on angiogenesis.

The data presented in the current study demonstrated that post-tMCAO WBV increases circulating serum-derived EVs containing irisin. At this juncture, we like to mention that circulating EVs released post-tMCAO WBV treatment likely contain more factors than only irisin, and future proteomic analyses are needed to characterize the contents of EVs. Further studies are also needed to determine the cellular origin and derivation of these EVs in order to confirm that are brain-derived. Given the fact that apart from the presence of irisin in EVs, post-tMCAO WBV also increases circulating irisin protein levels in the serum and cortical penumbra of middle-aged female rats. Overall, these data suggest a key role for irisin in WBV-conferred cognitive improvement. Although the mechanism by which irisin confers neuroprotection and improves cognition is not fully known, the hormone plays a crucial role in preserving mitochondrial function, mitigating oxidative stress, and elevating expression of BDNF, among other neuroprotective measures (Wrann, 2015). Irisin has a demonstrated role in the CNS, such as promoting neuronal proliferation through the STAT3 pathway (Moon et al., 2013), increasing synaptic plasticity through the upregulation of BDNF (Kim et al., 2019), and regulation of oxidative stress and the inflammasome (Peng et al., 2017; Wang et al., 2018). Our previous studies demonstrated that WBV therapy works in similar pathways, by increasing the expression of BDNF, reducing infarct volume, and reducing inflammasome protein levels in the brain (Raval et al., 2018). BDNF is a potent neuroprotective agent and post-stroke WBV-induced BDNF increase may be responsible for hippocampal neuronal survival after WBV and improved hippocampal-dependent cognition and anxiety-like behavior observed in the current study. In support of our findings, animals undergoing a chronic restraint stress (CRS) showed increased anxiety-like behavior and memory impairment, along with synaptic atrophy and neuronal degeneration. Interestingly, WBV reversed this behavioral dysfunction, inhibited the degeneration of neurons, alleviated the damage of neurons and the pathological changes of glial cells, enhanced trophic factor expression, and ameliorated the downregulation of dendritic and synaptic proteins after CRS. It was suggested that the effect of WBV in rats may be mediated via the reduction of hippocampal neuronal degeneration and by improving expression of synaptic proteins (Cariati et al., 2021; Peng et al., 2021). Post-stroke high-intensity interval training reduced ischemic brain damage and upregulated pTrkB (a major cascade of BDNF actions) and FNDC5 expression in the cortex of rats (Pin-Barre et al., 2021), which underscores its role in neuronal survival, hippocampal neurogenesis, synaptic plasticity, and functional recovery (Liu and Nusslock, 2018; Cohan et al., 2019; Luo et al., 2019). Therefore, future studies investigating the efficacy of post-stroke irisin treatment to improve mitochondrial and vascular functions, thus protecting the brain from ischemic damage and improving cognition, are needed.

Finally, the current study suggests that WBV improves post-stroke functional and cognitive deficits in middle-aged rats of both sexes and that irisin may be responsible for conferring the beneficial effects of WBV. While more studies are needed to characterize the role of irisin, based on data in the current study, WBV has a potential for clinical translation to improve post-stroke cognitive deficits. The most attractive aspect of low-frequency WBV therapy is that it could be implemented at an early stage as it is non-invasive and stress-free. In support of our approach, a study simulating helicopter-induced LFV/WBV immediately after tMCAO showed a significant reduction in infarct size and better neurological outcomes compared with control or actual helicopter-induced vibrations. Helicopter transportation is a common occurrence and will become routine with use of Tenecteplase, in which a “drip and ship” standard of care has been implemented (stroke patient receives tPA while transported by helicopter) (Rabinstein et al., 2020). Helicopters expose patients to several physical factors including LFV. Vibrations from helicopter transport showed no harm or benefit in this study. This study supports the early use and safety of LFV and justifies early LFV exposure for stroke patients (Dhanesha et al., 2020). Mindful of our ultimate goal to translate scientific discoveries into practices that improve health, we keep foremost in mind the importance of being realistic in trying to advance innovations in stroke care and prevention. What we develop must be feasible in practice. Effective interventions to reduce the risk and debilitating consequences of stroke in frail stroke patients also must be efficient, low-cost, and easily scalable. WBV platforms are widely available at low cost and are easy to implement even outside the clinics. WBV intervention could easily be tested in clinical settings and widely disseminated to community-based centers or in private homes where stroke patients receive care.

## Data availability statement

The original contributions presented in the study are included in the article/Supplementary material, further inquiries can be directed to the corresponding authors.

## Ethics statement

The animal study was reviewed and approved by the IACUC University of Miami.

## Author contributions

AR, HB, and WD: conceptualization, resources, and writing—review and editing. NK, HB, and AR: methodology. NK and JS: formal analysis. NK, AR, and JS: investigation. NK, WM, and OF-A: data analysis. NK and AR: writing—original draft preparation. All authors contributed to the article and approved the submitted version.
