# Supplementary material for: Post-stroke low-frequency whole-body vibration improves cognition in middle-aged rats of both sexes
Source: Front Aging Neurosci. 2022 Aug 17;14:942717. doi: 10.3389/fnagi.2022.942717 (PMC9428155; doi:10.3389/fnagi.2022.942717)
Supplement: Supplementary file 1 [file Data_Sheet_1.docx]

**Supplemental material**

Methods of Euthanasia: Rats were anesthetized with 4% isoflurane followed by tissue (brain/blood) collection for various biochemical analyses. In a separate cohort of rats, animals were anesthetized with 4% of isoflurane and a. median sternotomy was then performed in the anesthetized animal. The apex of the left cardiac ventricle is incised, and a closely fitting PE-240 polyethylene catheter with a slightly flared tip, filled to the apex with isotonic saline, was inserted through the ventricle into the root of the aorta and ligated in place. The descending aorta wa clamped with small hemostat forceps, and the tip of the right atrium was incised to permit egress of the perfusate. Perfusion begun with isotonic saline delivered under a constant pressure of 100-200 mmHg for 15 sec. The perfusate was then switched to 4% paraformaldehyde for 20 min at 100-200 mmHg pressure. At the end of perfusion, animals were decapitated, and brains were collected for histology.

**Table 1: Physiological Variables:** We monitored physiological parameters prior to and after tMCAO surgery and these data are presented as table 1. The results presented in table 1 show no significant variations in parameters under investigation. Data presented as mean ± SD.

| **Group** | **Variables** | **Middle-aged female** | | **Middle-aged male** | |
| --- | --- | --- | --- | --- | --- |
|  |  | **Before tMCAO** | **After tMCAO** | **Before tMCAO** | **After tMCAO** |
| **Sham** | Body weight (g) | 382 ± 40 | | 649 ± 65 | |
|  | Cranial Temp (°C) | 36.6 ± 0.16 | 37 ± 0.31 | 36.6 ± 0.1 | 37.24 ± 0.12 |
|  | Rectal Temp (°C) | 36.6 ± 0.20 | 37 ± 0.36 | 36.6 ± 0.11 | 37.28 ± 0.24 |
| **tMCAO** | Body weight (g) | 381 ± 62 | | 659 ± 72 | |
|  | Cranial Temp (°C) | 36.6 ± 0.15 | 36.9 ± 0.24 | 36.7 ± 0.1 | 37.28 ± 0.45 |
|  | Rectal Temp (°C) | 36.6 ± 0.12 | 36.8±0.28 | 36.7 ± 0.16 | 37.38 ± 0.48 |

**Figure 1: Blood pressure monitoring in middle-aged female:** Using the tail cuff method (CODA 2 system; Kent Scientific), hemodynamic measures were evaluated. Ten measurements were taken for each animal for five consecutive weeks and the mean value was reported as supplemental Figure 1. These parameters include measurements for systolic pressure, diastolic pressure, and average blood pressure. Data presented as mean ± SD. Clear bar indicates Sham + WBV, black bar indicates tMCAO + NO-WBV and red bar indicates tMCAO + WBV.

Systolic mmHg

Week-1

Week-2

Week-3

Week-4

Week-5

Week-6

Baseline

Post-tMCAO

Diastolic mmHg

Week-1

Week-2

Week-3

Week-4

Week-5

Week-6

Baseline

Post-tMCAO

Mean BP mmHg

Week-1

Week-2

Week-3

Week-4

Week-5

Week-6

Baseline

Post-tMCAO

**Figure 2: Post-tMCAO WBV therapy reduces infarct volume in middle-aged rats of both sexes:** Rats treated with either WBV or No-WBV after tMCAO survived for a month followed by histopathological assessment of the brains as described (Raval et al., 2018). Rats were anesthetized and perfused via the ascending aorta with 4% paraformaldehyde for 20 min after first being perfused for 2 min with saline. The rat heads were immersed in FAM for 1 day before the brains were removed. The brains were kept in FAM at 4^o^C for at least 1 additional day, and then coronal brain blocks were fixed in paraffin. All brains were cut into 10-μm thick sections from 5.1 mm to -7.5 mm from bregma at 9 standard levels to span the entire infarcted area. Sections of the 9 levels (bregma levels 5.2, 2.7, 1.2, -0.3, -1.3, -1.8, -3.8, -5, -7.3) were stained with hematoxylin and eosin to visualize the infarcted areas and to calculate infarct volumes. The electronic images of the tissue sections were obtained using a CCD camera and infarct volume was quantified using an MCID image analysis system (Raval et al., 2018). Quantification of infarct volume demonstrated that, compared to control (No-WBV) rats, WBV treated rats displayed a significantly reduced infarct volume in male rats. In the female No-WBV group, tMCAO resulted in 354 ± 145 mm^3^ (n = 6; p<0.05), while WBV yielded 191 ± 182 mm^3^ (n = 3) of infarct volume. We have previously demonstrated that mild (60 min) tMCAO followed by WBV therapy (twice a week for a month) significantly reduces infarct volume in reproductively senescent female rats (Raval et al., 2018). The observed difference in ischemic protection mediated by WBV may be due to difference in tMCAO timing, 60 min versus 90 min of tMCAO in the current study. In the male rat No-WBV group tMCAO resulted in 254 ± 88 mm^3^ (n = 5; p<0.05), while WBV yielded 98 ± 54 mm^3^ (n = 5) of infarct volume.


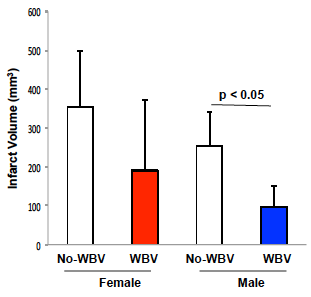


**Figure 3:** Expression of systemic inflammatory proteins in middle-aged female rats 24 hours post tMCAO. Mean, standard deviation, and p-values of serum inflammatory proteins analyzed using the Bioplex assay 24 hours post tMCAO in middle-aged female rats. P values represent significant differences between sham vs. tMCAO groups. *p<0.05, **p<0.01, ****<0.001

**
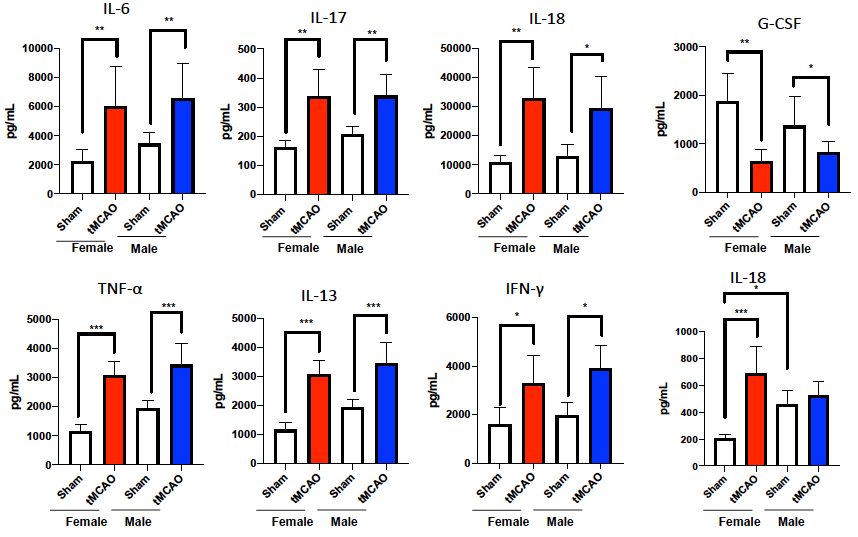
**

**
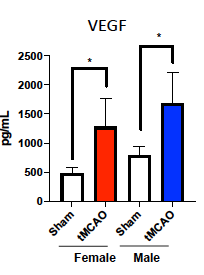
**

**Table 2:** Expression of systemic inflammatory proteins in middle-aged female rats 24 hours post tMCAO. Mean, standard deviation, and p-values of serum inflammatory proteins analyzed using the Bioplex assay 24 hours post tMCAO in middle-aged female rats. P values represent significant differences between sham vs. tMCAO groups. Green indicates up-regulation and red indicates down-regulation.

| **Analyte** | **Sham** | **tMCAO** | **p value** |
| --- | --- | --- | --- |
| IL-1α | 774.8 ± 451.4 | 836.8 ± 316.1 | >0.99 |
| Il-1β | 203.4 ± 32.65 | 687.3 ± 198.0 | 0.017 |
| IL-2 | 9232 ± 4244 | 21603 ± 7224 | 0.003 |
| IL-4 | 641.9 ± 242.5 | 938.1± 516.8 | 0.682 |
| Il-5 | 1687 ± 227.0 | 2784 ±1328 | 0.047 |
| Il-6 | 2231 ± 807.9 | 5997± 2788 | 0.0137 |
| Il-7 | 980.6 ± 641.1 | 3023 ± 2658 | 0.9717 |
| IL-10 | 1511 ± 371.0 | 777.5 ± 157.6 | 0.002 |
| IL-12 | 3377± 1763 | 4331 ± 2452 | >0.99 |
| IL-13 | 1663± 598.4 | 863.3± 269.8 | 0.541 |
| IL-17 | 163.4± 21.86 | 337.0± 91.84 | 0.0136 |
| IL-18 | 10868± 2447 | 32915±10656 | 0.0144 |
| GM-CSF | 501.7± 155.7 | 148.3± 43.33 | 0.035 |
| G-CSF | 1880± 561.4 | 638.1± 243.5 | 0.003 |
| GR-KCO | 459.8± 36.48 | 991.5± 308.0 | 0.0927 |
| GR-KCO | 459.8± 36.48/ | 991.5± 308.0 | 0.0927 |
| IFN-γ | 1612± 681.1 | 3287± 1140 | 0.0465 |
| M-CSF | 179.2± 118.6 | 224.4± 125.6 | 0.9973 |
| MIP-1 | 181.7± 71.88 | 429.2± 109.3 | 0.0591 |
| RANTES | 2691± 1058 | 2241± 595.7 | 0.9576 |
| TNFα | 1159± 238.3 | 3068± 468.7 | 0.031 |
| VEGF | 480.0± 104.6 | 1278± 491.3 | 0.163 |

**Table 3:** Expression of systemic inflammatory proteins in middle-aged male rats 24 hours post tMCAO. Mean, standard deviation, and p-values of serum inflammatory proteins analyzed using the Bioplex assay 24 hours post tMCAO in middle-aged males rats. P values represent significant differences between sham vs. tMCAO groups. Green indicates up-regulation and red indicates down-regulation.

| **Analyte** | **Sham** | **tMCAO** | **Sham/tMCAO**  **p value** |
| --- | --- | --- | --- |
| IL-1α | 1086± 207.5 | 922.6±201.4 | >0.999 |
| IL-1β | 520.4± 105.3 | 493.8±88.49 | >0.999 |
| IL-2 | 13097± 1827 | 12517±955.8 | >0.999 |
| IL-4 | 835.4± 175.8 | 884.3± 83.46 | >0.999 |
| IL-5 | 2061± 182.0 | 2104± 80.05 | >0.999 |
| IL-6 | 3448± 793.7 | 6572± 2370 | 0.036 |
| IL-7 | 2233± 2522 | 4609± 7017 | 0.916 |
| IL-10 | 2233± 2522 | 4609± 7017 | 0.854 |
| IL-12 | 4477± 1123 | 4703± 1156 | >0.999 |
| IL-13 | 1957± 726.5 | 1418± 448.9 | 0.815 |
| IL-17 | 208.2± 24.33 | 338.9± 74.44 | 0.036 |
| IL-18 | 208.2± 24.33 | 338.9± 74.44 | 0.044 |
| GM-CSF | 680.9± 376.6 | 146.8± 24.74 | 0.004 |
| G-CSF | 1368± 605.7 | 816.6± 231.7 | 0.567 |
| GR KCO | 550.6 ± 178.3 | 1000 ± 543.4 | 0.225 |
| IFN-γ | 1951 ± 542.4 | 3909 ± 952.1 | 0.019 |
| M-CSF | 210.9 ± 64.08 | 273.9 ±64.37 | 0.973 |
| RANTES | 2226 ± 1011 | 1836 ± 427.9 | 0.973 |
| TNFα | 1933 ± 267.4 | 3436± 729.9 | 0.013 |
| VEGF | 792.4 ± 144.4 | 1688± 525.0 | 0.033 |

**Table 4:** Expression of systemic inflammatory proteins in middle-aged female rats 30 days post-tMCAO and WBV treatment. Mean, standard deviation, and p-values of serum inflammatory proteins analyzed using the Bioplex assay 30 days post tMCAO and 30 days of twice daily WBV treatment in middle-aged female rats. P values represent significant differences between tMCAO + WBV vs. tMCAO + NO- WBV groups. Green indicates up-regulation and red indicates down-regulation.

| **Analyte** | **tMCAO +** | | |
| --- | --- | --- | --- |
|  | **WBV** | **NO-WBV** | **WBV /**  **NO-WBV**  **p value** |
| IL-1α | 703.2± 100.3 | 1003± 296.5 | 0.502 |
| IL-1β | 703.2± 100.3 | 1246± 341.6 | 0.023 |
| IL-2 | 15333 ± 1275 | 10511±1244 | 0.288 |
| IL-4 | 1218 ± 454.5 | 1169± 95.45 | 0.997 |
| IL-5 | 2308 ± 430.5 | 2813 ±165.2 | 0.697 |
| IL-6 | 3057 ± 831.2 | 5200 ± 955.2 | 0.046 |
| IL-7 | 1847 ± 287.3 | 1722 ±820.5 | 0.999 |
| IL-10 | 1431 ± 393.4 | 1538 ±485.3 | 0.997 |
| IL-12 | 5599 ± 1571 | 8538 ± 1722 | 0.027 |
| IL-13 | 1950± 604.7 | 952.1±362.4 | 0.338 |
| IL-17 | 249.9± 45.75 | 548.7±161.1 | 0.001 |
| IL-18 | 19983± 2308 | 40462±18510 | 0.007 |
| GM-CSF | 1384 ± 55.72 | 963.9 ± 364.4 | 0.641 |
| G-CSF | 221.3 ± 33.71 | 211.4 ± 87.86 | 0.999 |
| GRO KC | 670.0 ± 66.71 | 622.6 ± 117.5 | 0.994 |
| IFN-γ | 4157± 401.9 | 6480± 1739 | 0.050 |
| M-CSF | 422.8 ± 137.3 | 543.9 ± 165.1 | 0.893 |
| MIP-1a | 255.7 ± 67.53 | 408.6 ± 157.4 | 0.732 |
| RANTES | 555.6 ± 87.33 | 748.6± 327.8 | 0.899 |
| TNFα | 4092 ± 1509 | 7466 ± 977.3 | 0.039 |
| VEGF | 1446 ± 147.1 | 880.7 ± 118.5 | 0.024 |

**Table 5:** Expression of systemic inflammatory proteins in middle-aged male rats 30 days post-tMCAO and WBV treatment. Mean, standard deviation, and p-values of serum inflammatory proteins analyzed using the Bioplex assay 30 days post tMCAO and 30 days of twice daily WBV treatment in middle-aged male rats. P values represent significant differences between tMCAO + WBV vs. tMCAO + NO-WBV groups. Green indicates up-regulation and red indicates down-regulation.

| **Analyte** | **tMCAO +** | | |
| --- | --- | --- | --- |
|  | **WBV** | **NO-WBV** | **WBV /**  **NO-WBV**  **p value** |
| IL-1α | 510.4± 44.67 | 983.4 ±199.3 | 0.0510 |
| IL-1β | 415.0 ± 77.62 | 1002 ± 239.3 | 0.0018 |
| IL-2 | 6714 ± 1111 | 13121 ±3845 | 0.0018 |
| IL-4 | 456.2 ± 63.64 | 821.3 ±394.8 | 0.5527 |
| IL-6 | 2822 ± 511.4 | 5011 ± 1249 | 0.027 |
| IL-7 | 576.6 ± 101.8 | 2076 ± 941.9 | 0.692 |
| IL-10 | 1017 ± 204.1 | 565.0 ±95.98 | 0.042 |
| IL-12 | 2444 ± 297.9 | 4647 ± 1465 | 0.461 |
| IL-13 | 927.7 ± 94.36 | 1573 ± 889.3 | 0.742 |
| IL-17 | 255.7 ± 107.2 | 353.4 ±123.3 | 0.713 |
| IL-18 | 9735 ± 2658 | 19494± 7121 | 0.049 |
| G-CSF | 72.18 ± 10.99 | 227.3 ±91.08 | 0.051 |
| GM-CSF | 727.7 ± 193.8 | 2008 ± 338.8 | 0.001 |
| GRO-KC | 612.1 ± 131.9 | 957.9 ±122.6 | 0.001 |
| IFN-γ | 2603 ± 784.8 | 4659 ± 1439 | 0.047 |
| M-CSF | 261.6 ± 83.70 | 343.3 ±72.44 | 0.684 |
| MIP-1 | 90.57± 15.27 | 391.8 ±177.6 | 0.192 |
| RANTES | 1301± 194.3 | 1237 ± 293.5 | 0.999 |
| TNFα | 1876 ± 425.2 | 4515 ± 1080 | 0.008 |
| VEGF | 1143 ± 354.5 | 530.0 ±156.3 | 0.039 |
